# Learning From Elite Athletes’ Experience of Depression

**DOI:** 10.3389/fpsyg.2018.02062

**Published:** 2018-10-26

**Authors:** Florence Lebrun, Àine MacNamara, Sheelagh Rodgers, Dave Collins

**Affiliations:** ^1^Institute of Coaching and Performance, School of Sport and Wellbeing, University of Central Lancashire, Preston, United Kingdom; ^2^Grey Matters Performance, Ltd., Stratford-upon-Avon, United Kingdom

**Keywords:** elite sport, mental health issues, symptoms, genesis, stigma

## Abstract

Sport has become increasingly aware of the challenges associated with Mental Disorders and, to a greater extent, mental health issues (MHIs). This study sought to explore in depth the subjective experiences of elite athletes who suffered from a specific clinical MHI, namely depression. In addition, we explored their perceptions of the prevalence and stigma surrounding MHIs in elite sport environments. Data were collected from four elite athletes (1 female and 3 males; 2 team and 2 individual sports) using semi-structured interviews and analysed using Interpretative Phenomenological Analysis. Participants described both sport and non-sport related triggers in regard to their depression (e.g., institutional mistreatment and bullying, injuries, previous MHI, and miscarriage), as well as a range of behaviour, performance (e.g., decline), and personality changes compared to their normal functioning. Although the participants stressed the widespread prevalence of MHIs in elite sport, they also acknowledged the stigma associated with MHIs in high-level sport environments and its detrimental role for athletes. This study has important practical and diagnostic implications, and highlights the need for further research to assess the extent and scope of clinical MHIs in sport as well as to inform the care for this unique population.

## Introduction

Although high-profile performers are increasingly open to talking about their experience of mental health issues (MHIs) (Arvinen-Barrow, 2016), suffering from MHIs is still stigmatized in many sports (Bär and Markser, 2013; Rice et al., 2016; Roberts et al., 2016; Sebbens et al., 2016). This is especially so for elite athletes, often depicted in the media as strong and powerful (Arvinen-Barrow, 2016) and, as a consequence, perceived as role models by the general public (Markser, 2011). In an effort to understand and proactively support athletes with MHIs such as depression, it is essential to understand the nature and development of such a MHI, as well as the way they experienced it. In this regard, we highlight three areas concerning depression in elite sport which need further examination – namely (1) its genesis, (2) its diagnosis and symptomatology, and (3) its prevalence and associated stigma.

Before proceeding, however, it is important to delineate the scope of our investigation and, indeed, the different issues which may currently be conflated by researchers, coaches, athletes and the media. The difference between mental health problems and a clinical disorder requires the assessment of numerous factors, including one’s level of distress and impairment in functioning (American Psychological Association [APA], 2013). In the following, the term “mental health issues” refers to the definition of mental disorders as provided by World Health Organization [WHO] (2018), namely: “Mental disorders comprise a broad range of problems, with different symptoms. However, they are generally characterized by some combination of abnormal thoughts, emotions, behaviour and relationships with others.”

The prevalence of MHIs, amongst both the general and sporting populations, has recently received considerable attention. In the United Kingdom, 25% of the population will experience a MHI at some point in their life (Mental Health Foundation, 2015), with depression currently affecting more than 300 million people over the world (World Health Organization [WHO], 2018). It is, therefore, not surprising that depression is also the most common and widespread MHI within elites (Doherty et al., 2016) and, particularly, retired athletes (Park et al., 2013). As the genesis of a MHI is a highly individualised and multidimensional process (Gouttebarge et al., 2016), Reardon and Factor (2010) suggested that, when it comes to elite athletes, “*depression might have nothing to do with their athletic pursuits or the athletic pursuits could be their way of coping with depression, or it even could be caused by athletic participation*.” (p. 963). Notably, no causal link between elite sport participation and depression has yet been established (Reardon and Factor, 2010).

While investigating the prevalence of depression within elite performers, research seems to have, so far, failed to consider that the symptoms encountered by elite athletes might differ compared to the general population (Bär and Markser, 2013; Doherty et al., 2016). Furthermore, when this is considered alongside the stigma about depression and a sporting culture that rewards toughness at any cost (Uphill et al., 2016), it is perhaps unsurprising that understanding about the prevalence and the genesis of depression encountered by elite athletes remains blurred and unclear (Roberts et al., 2016; Uphill et al., 2016). This line of research is important since scientific knowledge about the prevalence and genesis of depression in elite sport is still limited (Gouttebarge et al., 2015). In this regard, it may be that the uniqueness of the performance environment in which elite athletes operate influences their experience, the symptoms expressed, and their reactions to depression (Doherty et al., 2016). These differences add a layer of complexity to recognising, diagnosing, and estimating the prevalence of MHIs met by elite athletes. Previous research has started to identify the breadth, complexities, and heterogeneities of MHIs in elite sport (e.g., Schaal et al., 2011; Doherty et al., 2016; Rice et al., 2016; Lebrun and Collins, 2017). As such extending these findings, and understanding the extent to which the experience of elite athletes differs from the general population, is an important avenue for exploration.

In addition, the lack of physical and observable symptoms of depression limits the extent to which athletes acknowledge MHIs, such as depression, as a medical condition and may lead them to consider those symptoms as a sign of weakness or personal flaws (Delenardo and Terrion, 2014). A lack of information regarding the impact of psychological health on athletic performance might also inhibit athletes’ help-seeking behaviours (Gulliver et al., 2012). This reluctance to seek help might be especially true given the stigma, shame, and embarrassment associated with depression, as well as the desire to avoid *appearing* weak or vulnerable within the sporting environment (Gulliver et al., 2012; Delenardo and Terrion, 2014). There is, therefore, a need to proactively provide tools and resources to athletes and their support team that destigmatize MHIs in general, and depression in particular, together with an environment that encourages athletes to seek help (Gulliver et al., 2012).

Reflecting these various issues, the aim of this study was to examine the experiences of elite athletes who suffered from depression during their athletic career. Depression, as the most reported MHI in both general (World Health Organization [WHO], 2018) and sports populations (Schaal et al., 2011; Rice et al., 2016), offers a good vehicle to examine athlete experiences, their perceptions of how this is viewed in their environments, and offer some “compare and contrasts” with the general population picture. Semi-structured, qualitative interviews were conducted to explore and generate an in-depth understanding of participants’ “at the time” perceptions and *post hoc* reflections on their experiences of depression. Finally, we wanted to compare and contrast athletes’ experiences of depression with the well-documented picture in the general population.

## Materials and Methods

### Participants

According to Swann et al. (2015), “successful elite athletes not only compete at the highest level, but have experienced some success at that standard (e.g., winning an event or a medal)” (p. 11). Following their definition, four participants – two current and two recently retired “at the time of the interview” British elite athletes (1 female and 3 males; 2 team and 2 individual sports; *M* = 33*, SD* = 4.82) – were purposefully sampled to participate in this study on the basis that they had experienced depression, a formally assessed and diagnosed MHI(s) (as defined by the World Health Organization) during their athletic career. As an additional criterion, participants had to be either free of ongoing MHI(s) or have their condition safely under control.

### Procedure

Ethical approval was granted from the authors’ Institutional Ethics Committee. Athletes meeting the inclusion criteria were first contacted via e-mail through a network of personal contacts. All participants were provided with an informed consent form prior the start of their interview and were reminded that they could decide not to answer or withdraw from the project at any stage up until interviews had been anonymised. Data were collected by means of semi-structured interviews (Smith and Osborn, 2007). In order to ensure a degree of uniformity between the interviews, an interview guideline was developed specifically for the purpose of this study. The one-on-one interviews lasted approximately 90 min (e.g., respectively 91′50″, 83′20″, 97′05″ and 84′45″) and were carried out face-to-face by the first author. Interviews were audio-recorded and transcribed verbatim.

#### Graphic Timeline

**FIGURE 1:**
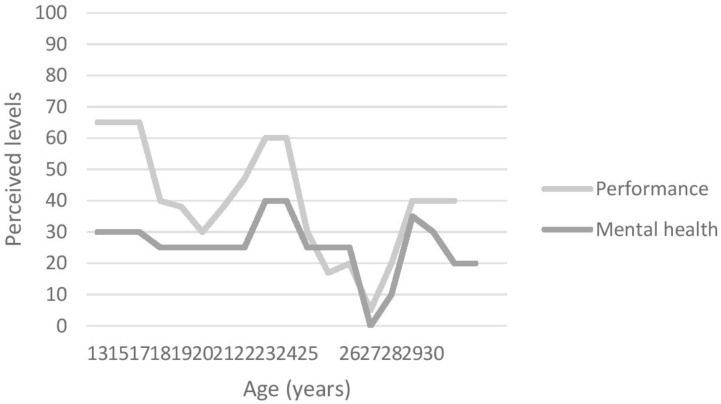
Participant 1’s graphic timeline.

**FIGURE 2:**
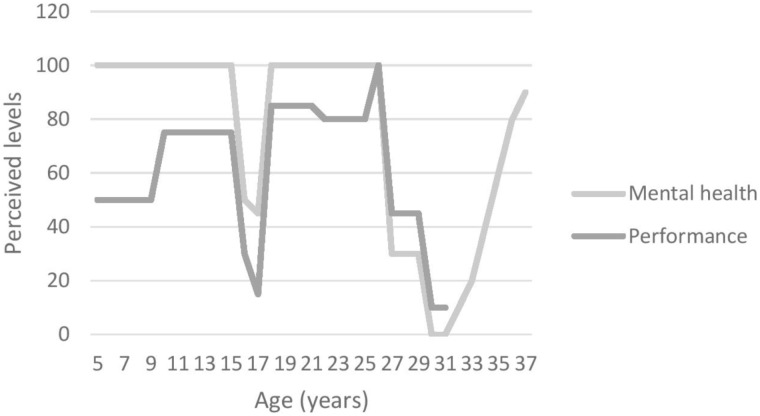
Participant 2’s graphic timeline.

**FIGURE 3:**
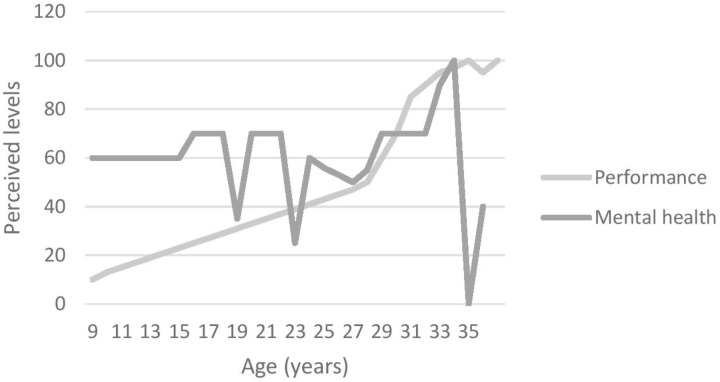
Participant 3’s graphic timeline.

**FIGURE 4:**
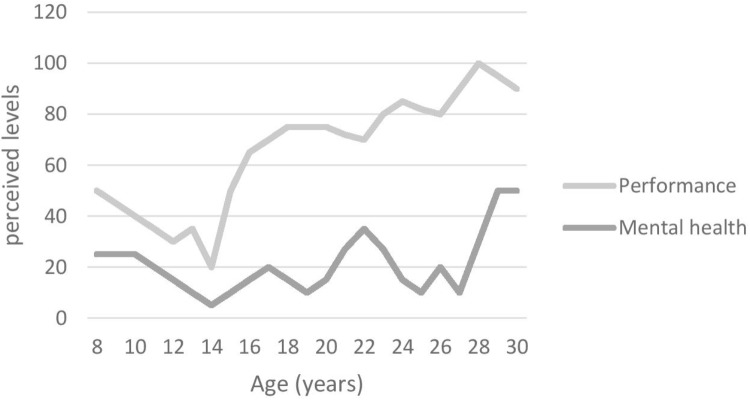
Participant 4’s graphic timeline.

At the start of the interview, participants were asked to draw a graphic timeline detailing reporting important events related to (1) their mental health issues(s) and (2) their sporting career. This aided recall method helps participants to overcome some memory decay inherent to the retrospective recall of specific times (Drasch and Matthes, 2013). Using landmarks events improves precision of recall regarding dating and the characteristics of those events (Drasch and Matthes, 2013). Those graphs (see Figures 1–4) were used by both interviewer and interviewee as a reference point through the whole interview.

#### Interview Guide

Using the graphic timeline as a prompt, the interview guide was used to lead the discussion while allowing participants to freely share their storeys (Jones et al., 2013). The questions avoided the use of jargon, were open-ended, and were framed as simply as possible using examples to clarify the questions if needed. The interview started with a first question asking about the participants’ background. This preamble was followed by various questions covering three main (i.e., diagnosis, genesis, symptomatology) and two secondary (i.e., stigma, incidence) areas designed to gain insight into athletes’ mental health history and perception. Probes and prompts were used to encourage participants to develop their answers, deepen the information shared and clarify relevant points (Jones et al., 2013) while at the same time allowing some flexibility to expand on issues raised by the participants.

#### Pilot Study

In development, pilot interviews were conducted with two athletes (two males; mean age = 33, σ = 5.657) matching the study inclusion criteria. These participants were asked to give feedback regarding the overall content of the interview in order to modify as appropriate and improve the interview guide created specifically for this study. Following the pilot study, participants’ feedback were positive about the content and format of the interview. The most notable modifications made by the first author to the interview guide was the inversion of two questions in terms of the flow of the interview.

### Data Analysis

Interpretative Phenomenological Analysis (IPA) aims to provide rich, descriptive accounts of how people perceive and make sense of their own lived experience (Smith, 2011) and was, therefore, the method chosen to analyse each narrative in detail and to examine in–depth each participant’s subjective experience in order to understand “*what it is like, from the point of view of the participants*” (Smith and Osborn, 2007, p. 53).

Data analysis was drawn on Smith and Osborn (2007)’s step-by-step approaches to IPA. Each transcript was first read multiple times along with the original recordings in order to become as familiar as possible with their content (Smith and Osborn, 2007). During this stage, researcher’s thoughts, observations, and other comments were annotated in the left margin (Smith and Osborn, 2007). Meaningful units reflecting participants’ quotes were then highlighted and the revision tools on Word (e.g., comment button) was used to comment and title initial themes (Smith and Osborn, 2007). Those initial themes and comments aimed to capture the essence of the meaningful units found in the text while taking into account the researchers’ interpretation (Smith and Osborn, 2007). This process was repeated through the whole transcript until all initial meaningful units were coded into themes (Smith and Osborn, 2007). An inventory of the generated themes was then realised and similar themes were clustered together (Smith and Osborn, 2007). Clusters were given a name and represented a higher order theme. The themes were then organised hierarchically providing an overall structure to the data set (Biggerstaff and Thompson, 2008). A table summarising the higher order themes was realised and this procedure was repeated with each transcript (Smith and Osborn, 2007). Once each transcript had been analysed, patterns across the transcripts were searched and a list combining all the themes contained in every transcripts was produced (Biggerstaff and Thompson, 2008). A particular focus was on the divergences and convergences between the transcripts (Smith and Osborn, 2007). Finally, a last table was created with all higher order themes (see Table 1).

**Table 1:** Participants’ profile.

Participant	Diagnosi(e)s	Triggers	Symptomatology	
1		*Sport-related trigger*	*Diagnostic criteria (DSM-V)*	*Other symptoms reported*
	Depression Anxiety ADHD	Bullying and mistreatment within a sport organisation	Depressed mood (e.g., feels sad, empty, hopeless). Markedly diminished interest or pleasure in all, or almost all, activities. Significant weight loss when not dieting or weight gain, or decrease or increase in appetite. Fatigue or loss of energy nearly every day. Feelings of worthlessness or excessive or inappropriate guilt. Recurrent thoughts of death, recurrent suicidal ideation without a specific plan, or a suicide attempt or a specific plan for committing suicide. The symptoms cause clinically significant distress or impairment in social, occupational, or other important areas of functioning.	Change of character Behavioural changes such as: At-risk behaviour Aggressiveness Increased impulsivity Disengaging/isolating themselves from others Withdrawing behaviour Increased anxiety Negative impact on performance

2		*Sport-related trigger*	*Diagnostic criteria (DSM-V)*	*Other symptoms reported*
	Depression	End of career due to injuries	Depressed mood (e.g., feels sad, empty, hopeless). Markedly diminished interest or pleasure in all, or almost all, activities. Fatigue or loss of energy nearly every day. Feelings of worthlessness or excessive or inappropriate guilt. Recurrent thoughts of death, recurrent suicidal ideation without a specific plan, or a suicide attempt or a specific plan for committing suicide. The symptoms cause clinically significant distress or impairment in social, occupational, or other important areas of functioning.	Change of character Behavioural changes such as: Aggressiveness Disengaging/isolating themselves from others Withdrawing behaviour Increased anxiety

3		*Non-sport-related trigger*	*Diagnostic criteria (DSM-V)*	*Other symptoms reported*
	Depression Anxiety	Miscarriage	Depressed mood (e.g., feels sad, empty, hopeless). Markedly diminished interest or pleasure in all, or almost all, activities. Fatigue or loss of energy nearly every day. Feelings of worthlessness or excessive or inappropriate guilt. Diminished ability to think or concentrate, or indecisiveness. The symptoms cause clinically significant distress or impairment in social, occupational, or other important areas of functioning.	Behavioural changes such as: At-risk behaviour (e.g., self-harm) Disengaging/isolating themselves from others Increased anxiety – worst case scenarios Negative impact on performance

4		*Non-sport-related trigger*	*Diagnostic criteria (DSM-V)*	*Other symptoms reported*
	Depression Anxiety (OCD)	Obsessive and Compulsive Disorder (OCD)	Depressed mood (e.g., feels sad, empty, hopeless). Markedly diminished interest or pleasure in all, or almost all, activities. Significant weight loss when not dieting or weight gain, or decrease or increase in appetite. Feelings of worthlessness or excessive or inappropriate guilt. Insomnia or hypersomnia (sleep disturbance). Fatigue or loss of energy nearly every day. Recurrent thoughts of death, recurrent suicidal ideation without a specific plan, or a suicide attempt or a specific plan for committing suicide.	Behavioural changes such as: At-risk behaviours Aggressiveness Increased impulsivity Negative impact on performance

*Summary of main themes and sub-themes for each participant.*

Anonymised quotes highlighting each theme are presented in the results section and stress the participants’ individuality as well as the theoretical convergence or divergence between their interviews (Smith and Osborn, 2007). For the sake of confidentiality and anonymity, no information enabling participants’ identification (e.g., sport, club name, etc.) were reported and numbers were given to the participants.

#### Trustworthiness

Peer-debriefing, direct quotes (Jones et al., 2013) and member-reflection (Smith and McGannon, 2018) were the methods used to ensure the trustworthiness of our findings. Peer-debriefing occurred through the research team’s feedback to the first author over the course of the research and, in particular, during the data analysis (Jones et al., 2013). The research team was used for critiques and challenges. The second author, for example, an experienced qualitative researcher, independently reviewed the first author’s data analysis, coding, and interpretation. The first and second authors, then, openly discussed the grouping of codes into them with the second authors challenging the first one until a consensus between the two was found. Subsequently, the third and fourth authors acted as critical friends and reviewed the final codes. Following Jones et al. (2013)’s suggestion, peer-debriefing was used in order to help guide the analysis and interpretation Following the cross analysis between the four transcripts and the final thematic structure, and in order to support the peer-debriefing process (Jones et al., 2013), the researchers asked each participant to give feedback on the themes generated from their own transcript, as well as to reflect and comment on a summary of the key findings of the study. Member reflection provides participants and researchers with an opportunity to engage in a dynamic process in order to explore what and highlight any gaps, similarities, contradictions or differences between the researchers’ and participants’ understanding of their accounts (Smith and McGannon, 2018). Only one participant engaged actively in this process and offered reflections on the report of their position. The researchers, at that point, collectively discussed the final findings and thematic structure until a consensus was reached. The results were, then, re-discussed, reviewed once again, and rewritten by all authors. Finally, the use of thick description by the means of direct quotes (Jones et al., 2013) provided a rich source of information to the reader without being tempered/contaminated by the authors’ interpretation.

## Results

The aim of this study was to explore the unique experiences of elite athletes who suffered from depression, as well as to examine their perception of the prevalence and stigma surrounding MHIs in elite sport environments. All participants were suffering from a range of symptoms causing distress and/or functioning impairment severely enough to have been diagnosed either by their general practitioner (GP; *n* = 2) or by a psychiatrist (*n* = 2) with depression. In addition, three of them also presented comorbidities (e.g., including attention deficit/hyperactivity disorder [ADHD; *n* = 1), anxiety (*n* = 3) and obsessive and compulsive disorder (OCD; *n* = 1)]. Figures 1–4 illustrate participants’ perception of their levels of mental health and performance over time. Those figures have the advantage of offering a broad overview of their mental health and performance fluctuations before, during, and after their episode of clinical depression.

As the aim of this study was to understand how elite athletes’ experienced and viewed their condition, participants were asked about their perceived triggers of their depression and their symptomatology. Following this, participants’ own perceptions of how MHIs are viewed within their sport environments are presented. Finally, in the general discussion section, we highlight some key similarities and differences between elite athletes and general population, using this to present some applied implications and areas for further study.

### Triggers

Although the development of depression is a multidimensional process with multiple factors in action (e.g., biological, social, socio-economical, and environmental; Gouttebarge et al., 2016), participants made sense of their experience and conceptualised the aetiology of their depression by identifying a variety of triggers that had precipitated, or could have precipitated, their depression. For two participants, the main triggers were linked to their sport participation (e.g., injury, sport organisation); whereas for the other two participants the issue had nothing to do with their athletic pursuit (Reardon and Factor, 2010) and was identified as non-sport but life-related factors (i.e., miscarriage, OCD).

#### Sport-Related Triggers

Participants 1 and 2 were strong in their belief that their depression was caused by their participation in elite level sport. The institutional mistreatment and bullying experienced by Participant 1 were, for example, identified as the main triggers for his depression. He further claimed that the sole focus of his sport organisation was on performance excellence which negatively impacted his physical health and well-being (cf. Miller and Kerr, 2002). Illustrating this, Participant 1 described how he:

…got put under so much pressure and I think ethically it was completely wrong. (…) They caused me problem after problem. As bad as me saying that, but generally, wholeheartedly they caused my deterioration in health (…) They were awful. They would devalue what I was doing and then expect me to go and perform (…) I was an inconvenience to have a personality.

Participant 2 conceptualised his depression as triggered by serious injuries causing his involuntary retirement from sport which had a number of social, financial, and identity-related consequences. Reflecting this, he described how he:

… got forced into retirement through serious injuries (…) [sport] is the only job I’ve ever had since leaving school which meant I lost my job, I lost my career (…) I’ve always been a [sport] player, that’s my identity and I lost it. I didn’t know if I belonged anymore (…) and when I got forced into retirement I lost thirty best mates.

Transition to retirement is known as a high risk period for depression and emotional distress especially when leading to a loss of identity (Park et al., 2013). Furthermore, and in addition to the loss of athletic identity, losing his providing role for his family was emotionally difficult for Participant 2: “*I don’t miss playing, I don’t miss the [sport] side. That doesn’t bother me. It were just not being able to support me family. That’s when I couldn’t get the thoughts of suicide out of my mind*.”

#### Non-sport Related Triggers

In contrast to the sport-related triggers described by Participants 1 and 2, Participant 3’s miscarriage was described as the major life event precipitating her depression (e.g., “*That was because I had a miscarriage in the Christmas yeah, and then that makes it all, that’s the worst time of year because it’s all about families at Christmas*.”). Participant 4 identified his pre-existing OCD as the main factor leading to his depression.

I remember being depressed for a long time so I don’t really remember sort of having OCD and then being depressed. I remember the last time when it was diagnosed it was the OCD was caused depressive disorder. I’d say that OCD isn’t particularly negative in my life, apart from the fact that it makes me depressed or has made me depressed. And still does at times make me depressed.

### Symptomatology

Even though all the participants reported different triggers precipitating their depression, it was expected that they would report and share common symptoms underpinning the diagnosis of such a MHI (cf. American Psychological Association [APA], 2013, p. 161). A brief overview of each participant’s subjective experience of depression, including the symptoms reported, is summarised in Table 1. This is, of course, not meant to represent the true complexity and heterogeneity of those experiences. Discriminating between depressive symptoms and “normal” reactions or feelings to adverse events can be as difficult (Cornford et al., 2007; Gulliver et al., 2012) for athletes, as with the general population, and requires the assessment of a range of elements, including the individual’s level of distress and impairment (American Psychological Association [APA], 2013). However, the participants described how symptoms or warning signs, for example behavioural changes, “personality” changes, and performance decrements (see Table 1) manifested themselves over time. For Participant 1, one of the most difficult aspects was, for example, the feeling of being out of control and looking for a way-out (e.g., drugs or self-harm):

So it manifests in certain behaviours like things I would do, you’re just like so out of character. Like my impulsive nature, I couldn’t control, I was just like, ‘oh I need some help because I am out of control.’ (…) I wasn’t happy and I didn’t feel anything. And so you’d go chasing sensations and stuff like that. I talked about doing heroin during that time because I was just like I can’t live in this, in this state (…) I broke up with my girlfriend because I was basically cutting all ties with everyone. So that, to make it easier for me to just sever it and just go.

Like Participant 1, Participant 4 was looking for an escape but in his case his thoughts were about making life changes and self-harm, which were an aspect of his OCD, were intensified by his depression:

Lower energy, tired, don’t like doing things, just feel irritable, hungry all the time, don’t sleep particularly well, always looking to change something like I could change jobs or something like that you know (…) My compulsion would be to think about self-harm and think about suicide.

While Participants 1 and 4 mostly reported behavioural changes, Participant 2 also described what he perceived as a “change of personality”:

Just my personality had completely changed. When I was suffering it just wasn’t me, it wasn’t my personality (…) I don’t know, my wife just said it was just, you didn’t take anyone, didn’t take us on. You weren’t speaking. You weren’t doing anything. You used to just sit watching the telly and there were just no expression in my face (…) I used to get upset at the littlest thing and stuff like that.

In addition to behavioural and “personality” changes, participants also mentioned performance decrements as a symptom or sign of their depression. Figures 1–4, for example, illustrate participants’ perceived relationship between their mental health and performance. The impact of MHIs on productivity and output might be more obvious for athletes than the general population. Notably, however, the relationship is complex and idiosyncratic. When looking back at his performance at the time of his depression, Participant 1 identified performance decrements and remembered that “*It [performance] was dropping and dropping and (…) I wasn’t getting any help or any coaching input.”* Unfortunately, little attention was paid by others (e.g., coaches, peers, family) to his dramatic drop in performance. Participant 3 also recognised that her mental health and well-being had an impact on her performance outcomes, although her performance decline was perceived as modest compared to Participants 1 and 2 – as she explained “*I think if I was happier, then, I would perform better*. (…) *it’s hard to get your best performance when you don’t feel happy.”* Participant 4, however, was somewhat of an outlier in that his level of performance was not closely linked to his mental health. He acknowledged, however, that:

… sometimes it would affect performance, sometimes it wouldn’t. It would affect how you train. So when you’re feeling low sometimes your training isn’t very good you don’t train very well you don’t practise very well. But I wouldn’t say it would affect my match performance really.

Interestingly, in spite of their symptoms and the extent to which depression impacted on their overall functioning and sport performances, Participants 1, 3 and 4 continued to train and compete at the highest level, while Participant 2 tried a “comeback” after his recovery from injuries despite the fact that he was, at the same time, suffering from depression. Although elite athletes frequently continue to “more or less” function in their sport by training and performing whilst trying to hide their distress (Doherty et al., 2016), a difference was noticeable between those whose triggers were sport-related (i.e., Participants 1 and 2) and those whose triggers were not sport-related (i.e., Participants 3 and 4). In this regard, Participants 1 and 2 described how their depression negatively impacted on their passion and/or motivation for sport. Illustrating this, Participant 2 revealed that:

I couldn’t be bothered training, I couldn’t be bothered, although I did train but I’d just turn up. Felt like I were just turning up. I wasn’t there because I was enjoying it. The only reason I carried on playing in 2011 is because I wanted to get some money in for my family (…) I was just doing it for the money. I was doing it for the wrong reasons.

In contrast, Participant 3 noted that training and competing had a positive influence on her well-being, and for her “*Training is like a release and, in every other situation, training’s always made me feel better and feel back to normal again (…) I did start training as soon as I could. And it did make me feel better.”* Participant 4 presented a mixed picture in this regard in that, even though he often lacked the motivation to train, in general training and maintaining participation in this sport helped him to feel better.

Sometimes I’m feeling low and then I go, we have like a training session makes me better afterward but sometimes you feel bad after training sometimes you feel good going for a session feel bad afterward so yeah (…) and the thing, like exercising in general can lift the mood so sometimes feeling very low and don’t want to train, don’t want to go and then finish afterward and just feel pretty good.

All these accounts support Doherty et al. (2016)’s argument that there is a need to look beyond the DSM-5 criteria when working with elite athletes, and to be cognisant of the context in which athletes operate. Furthermore, and in line with Reardon and Factor (2010)’s suggestion that athletic pursuit can precipitate, worsen a performer’s depression, or be a means to cope with a pre-existing condition, those last accounts illustrate how Participants 3 and 4 used their sport participation as a means to cope with their mental health fluctuations and depression (see Figures 1–4), while Participants 1 and 2 had to find other ways using a broad range of coping strategies to fight against their depression.

### MHIs Within Sport Environments

#### The Prevalence of MHIs in Sport

Participants described MHIs in general as being more common in elite sport than is usually portrayed. Participant 1 described how the prevalence:

… is a lot higher than most people would think. There was another player, she also had, and then there was another player who also had a problem. So you can see that then suddenly you go, ‘hold on a minute, all these people are having MHIs, what’s organisationally wrong with it.’

Supporting Participant 1’s opinion, when asked about the prevalence of MHIs in elite level sport, Participant 2 replied that they are “*very common just because of the amount of highs that we get. I know that 200 players have sought help. So it’s 23–25% of [sport] players.*” In line with these subjective accounts, Participant 3 also perceived the prevalence of MHIs as more common than it is recognised or generally accepted, at least in her sport environment:

It’s definitely more common than people think and I think most people don’t admit it if they are. Some admit it personally and some are in denial and some, I guess, just are depressed but don’t know and have never been diagnosed with it. (…) I know quite a few of them [other athletes] have been through the same as me. I can think of four or five at the moment that are in the same position.

Although all the participants perceived MHIs as prevalent in sport, and all knew other athletes suffering from MHIs, Participant 4 questioned the extent of the severity of those MHIs thus differentiating between sub-clinical and clinical levels of such issues as well as between their chronicity or acuteness.

I would say they’re very, very common, to the extreme which I have it or I’ve had it I don’t think so (…) a lot of teammates, a lot of former teammates have said ‘I’ve felt very low during this period or during this year, in that year’ and stuff like that so I think it’s very, very common but whether it’s a prolonged illness or whether it’s a short thing I don’t know.

#### The Perceived Stigma

Despite their prevalence in sport and in society in general, the stigma associated with MHIs was highlighted by the participants as a damaging factor in sport environments. Participant 2 explained why, in light of this stigma, he would not talk about his depression and why he now considers stigma as the main issue in sport:

… that stigma around there was there for me. I didn’t wanna tell anyone. I didn’t wanna tell the coaches in case they didn’t pick me which meant I’d have lost money for my family. Mental health, depression, that’s not the killer. The stigma is the killer because they don’t wanna speak about how they’re feeling (…) It’s a big taboo subject that people don’t wanna talk about if you ever mentioned mental health.

Supporting Participant 2’s view, Participant 4 explained that “*there can be a lot of stigma around mental health particularly for males.”* This stigma, often caused by a lack of knowledge and understanding regarding MHIs and depression, can be detrimental for the people suffering. False beliefs about depression, for example, led Participant 1’s physiotherapist to be afraid to work with him which, in turn, negatively impacted his knee rehabilitation:

So when we talk about my knee and rehabilitation I was at a disadvantage because of how he [the physiotherapist] felt. (…) He was like, ‘oh I can’t be left in a room on my own with him.’ Eight months later my next knee goes because I didn’t have the proper rehabilitation and that’s because of my mental health because I was blacklisted in terms of help for a bit.

Another false, yet widespread idea, often conveyed in sport environments and in general society, is that elite athletes are “mentally” tough and, therefore, should not show any sign of weakness (Gulliver et al., 2012). This culture had an impact on the participants. For example, it took a long time for Participant 4 to see a doctor or simply talk about his MHIs because of the embarrassment caused by these misconceptions “*I knew it wasn’t right but I was ashamed of it because it was perceived as weakness at the time.*” Although suffering from OCD and, therefore, also probably from depressive episodes since his childhood (see Figure 4), Participant 4 was formally diagnosed with depression by a psychiatrist for the first time at the age of 26. As a result of perceiving MHIs as a “mental” weakness, athletes tend to hide their problems as explained by Participant 2:

A lot of players used to think it was a weakness to ask for help. If you’re injured on a [sport] field you never show signs of weakness… the opposition will exploit you and they’ll run at you and they’ll target you.

When talking about their experience of depression within the elite environment, participants shared the common opinion that more education and resources about mental health is needed in order to fight the stigma surrounding this topic and the detrimental consequences those false beliefs can have on athletes’ mental health. As summarised by Participant 2:

I’ve come to learn is that depression and mental health has no boundaries. It doesn’t matter if you’re £5 an hour cleaner or £100,000 a week footballer. It doesn’t matter. It can get anyone so (…) Just to realise that you never, even though you’re a professional athlete you should never think that you’re immune to MHIs (…) It’s just like an injury. It’s just some of you can’t…you can’t help. You know, it’s not a flaw, it’s an illness. It can be treated, it can be fixed, it can be sorted out just like any other injury.

## Discussion

The present study aimed to broaden our understanding of elite athletes’ unique and subjective experience of clinical depression, their perceptions of how MHIs are viewed in elite level sport environments, as well as to highlight some of the key similarities and differences between elite athletes and people from the general population. Although comorbidities were observed (e.g., ADHD, anxiety, and OCD), depression was the common, clinically diagnosed MHI shared by all the participants. The present findings focused mainly on how the participants made sense of their experience by identifying the perceived main triggers behind the development of their depression, as well as the variety of symptoms they suffered from. Finally, and in spite of their apparent commonness (“*it is more common than we think*”), the stigma associated with MHIs in elite sport environments was perceived by the participants as tenacious and detrimental.

Whilst similarities exist between elite athletes and the general population regarding the diagnosis of depression and the symptoms encountered, dissimilarities were also noticeable. The triggers depicted by the participants as the cause behind the development of their depression were described as, either sport-related (e.g., forced retirement, institutional bullying and mistreatment) or life-related (e.g., miscarriage, previous MHI such as OCD). As such, elite athletes may face specific and sport-related triggering factors (e.g., Participants 1 and 2) as well as the other triggers found in the general population (e.g., Participants 3 and 4; Reardon and Factor, 2010). These findings support Reardon and Factor (2010)’s argument that depression can either be precipitated or worsened by a performer’s athletic pursuit, can be dealt with via sport participation, or not associated at all with their sport participation. These findings also further emphasise the importance of a person and context-centred approach to understanding MHIs, and are in line with previous studies highlighting severe injuries, major life events, comorbidities, career dissatisfaction (Gouttebarge et al., 2018), and career transition (e.g., retirement; Park et al., 2013) as risk factors of common mental disorders such as depression in elite sport.

Similarly, whilst the symptoms required for a diagnosis of depression in the general population (American Psychological Association [APA], 2013) were met by those athletes, other symptoms described by the participants diverged from the DSM-5 criteria. Beyond the usual set of criteria leading to the diagnosis of depression, athletes in the present study reported a mixed pattern of additional warning signs such as changes in behaviour (e.g., having at-risk behaviours, being out of control), in performance (e.g., performance drops, demotivation), and personality. This adds to Doherty et al. (2016)’s argument that there is a need to look beyond the DSM-5 criteria when working with elite athletes Although differentiating between signs of depression and “normal” range of reactions or feelings (e.g., sadness, tiredness) to adverse events can be difficult (Cornford et al., 2007; Gulliver et al., 2012), this argument is especially valuable when looking for signs or potential indicators of MHIs in athletes. In this regard, it might be useful to consider any accumulation of, and persistent changes in, behaviours and/or performance over time, as potential warning signs warranting further investigation (Hill et al., 2016) as well as the spectrum on which those issues might occur. Due to diagnostic issues, people easily consider that MHIs is a black-and-white issue. The distinction between an “issue” as such and a “disorder” *per se* is not easily discernible. Participant 4, for example, questioned the severity of MHIs met in sport environments – going from symptoms of mental health problems to mental health disorders *per se* – and, thereby, highlighted the necessity to understand and recognize the different levels of severity (spectrum) when it comes to MHIs. Unfortunately, to date, research about clinical depression – its’ extent, symptoms, and consequences - among elite athletes remains lacking. This dearth of information can partly be explained by the gap between the true prevalence of MHIs in sport and the treated (Henderson et al., 2013) or self-reported prevalence of MHIs (Gorczynski et al., 2017). More research on prevalence is warranted to properly assess the clinical prevalence of MHIs in elite sport, and elite athletes’ experiences of clinical and sub-clinical MHIs is needed in order to combat the stigmatisation surrounding such issues that still prevails.

The stigmatisation surrounding MHIs described in this study mirrors findings elsewhere in the literature (Gulliver et al., 2012). The false and internalised beliefs that elite athletes should be “mentally tough” and shouldn’t show any sign of weakness (Gulliver et al., 2012) are still dominant in elite sport environments and lead many athletes either to deny suffering from MHIs, or hide it because of the potential consequences of such a disclosure on their career (e.g., being excluded from the team or from a game; Hill et al., 2016; Uphill et al., 2016). The shame associated with MHIs and the necessity to hide any vulnerabilities from coaches, opponents, and teammates has previously been observed with male athletes (Doherty et al., 2016) and supports the idea that it is not expected nor accepted for elite male athletes to display any sign of weakness (Doherty et al., 2016). In addition to the absence of obvious identifiable medical signs of disease, this lack of knowledge and understanding - often cited as a barrier to help-seeking (Gulliver et al., 2012; Doherty et al., 2016) – may also explain the reluctance of athletes to consider depression as a proper illness instead of a personal flaw which, in turn, delays any help-seeking behaviours and treatment (McNair et al., 2002). As a result, and by the time athletes finally decide to seek for help, their condition has often moved from a subclinical to a clinical level of severity and impairment (Schinke et al., 2017).

Seeking help is a process involving different stages starting by becoming aware of the problem, followed by a perceived need for help and the identification of appropriate sources of help to access, and finally by the individual’s willingness to seek out and disclose their issue(s) to a potential source of help (Gulliver et al., 2010). It is, therefore, imperative to increase elite athletes’ and their entourage’s mental health literacy – namely “the ability to gain access to, understand, and use information in ways which promote and maintain good mental health” (Lauber et al., 2003, p. 248). Raising their level of knowledge and confidence to deal with those kind of issues are useful strategies in the prevention and management of MHIs (Sebbens et al., 2016). Those actions could also in parallel reduce the stigma associated with MHIs (Sebbens et al., 2016). Investigating and increasing elite athletes’ awareness of MHIs is essential to early detect and intervene on those issues as untreated they can often lead to more severe problems in terms of mental health and functional impairment but also in terms of performance issues (Markser, 2011; Gulliver et al., 2012).

The current study presents some strengths and limitations. One strength concerns the participants’ clinical diagnosis of depression by a GP or a psychiatrist compared to studies often resting upon self-reported measures (Gouttebarge et al., 2015; Gorczynski et al., 2017). Our research design allowed the exploration of participants’ experience of depression from their unique perspective in greater depth. As previously mentioned in the inclusion criteria, participants had either recovered from their MHIs by the time of the interview or had their condition safely under control. However, and despite some of the steps taken, such as the use of a graphic timeline to aid memory recall (Drasch and Matthes, 2013), to counter the limitations of retrospective research some memory decay may still have persisted and impacted on the results. In addition, some participants may have consciously or unconsciously omitted information due to the emotional difficulty and discomfort generated by the recollection of a distressful period of their lives. An additional element worth discussing is the potential influence of social desirability on participants’ account as participants may have been more willing to participate to this study due to their own interest in the present topic. Furthermore, and although a small sample of three participants is sufficient in allowing in-depth and detailed analysis of each participant’s data, specifically similarities and differences (Smith and Osborn, 2007), the authors would like to emphasise the difficulties encountered in the recruitment, difficulties previously highlighted by Glick et al. (2012) and mostly due to the main topic. The chosen population, the diversity of sport included (i.e., team and individual sports), the gender disparity and the sample size do, therefore, not enable a strict generalisation of the findings.

Finally, and in addition to the need for research that examines the true prevalence and symptomatology of MHIs in elite sport compared to the general population, future studies should also examine the coping strategies implemented by elite athletes to deal with such issues. Importantly, on their way to the top, elite athletes have to overcome a lot of challenges, using a set of different skills to face them. MHI(s) could, in this context, be viewed as an additional kind of challenge(s) that athletes might have to face at some point during their career or after. Our ongoing research is considering the extent to which athletes may often see a distinction between sport-related and other life challenges, leading them to not deploy, or feel helpless against, challenges such as MHIs. Research on coping strategies is, therefore, essential to the design of proactive interventions aiming to equip athletes with resources (e.g., knowledge and skills) to maintain their mental well-being or prevent as much as possible the development of clinical MHIs while taking the sport context and their unique needs in consideration.

## Ethics Statement

This study was carried out in accordance with the recommendations of UCLan ethics committee’s guidelines [BAHSS Ethics Committee, the University of Central Lancashire (UCLan)] with written informed consent from all subjects.

## Author Contributions

FL and DC were responsible for data acquisition while FL and ÀM were responsible for their analysis. FL and ÀM prepared a draft of the manuscript. SR and DC provided critical revisions to the final submitted version. ÀM and DC gave their approval for it to be published. Finally, all authors agreed to be accountable for all aspects of the work in ensuring that questions related to the accuracy or integrity of any part of the work are appropriately investigated and resolved and also involved in the conception and design of the work.

## Conflict of Interest Statement

ÀM, SR, and DC are all part of Grey Matters Performance, Ltd. The remaining author declares that the research was conducted in the absence of any commercial or financial relationships that could be construed as a potential conflict of interest.
